# Study of the Dynamic Development Law of Overburden Breakage on Mining Faces

**DOI:** 10.1038/s41598-020-63526-2

**Published:** 2020-04-16

**Authors:** Hongqing Zhu, Shuhao Fang, Yujia Huo, Jinlin Guo, Yan Wu, Lintao Hu

**Affiliations:** 1 School of Emergency Management and Safety Engineering, China University of Mining and Technology (Beijing), Beijing, China; 2 State Key Laboratory Coal Resources and Safe Mining, China University of Mining and Technology (Beijing), Beijing, China

**Keywords:** Coal, Geodynamics, Geology, Mineralogy, Petrology

## Abstract

Based on the geological conditions of overburden rock, the dynamic development law of overburden breakage was investigated by theoretical analysis and similarity model experiments in this paper. The formula of the compressive strength and No. ratio was obtained by testing the compressive strength of cylinder samples of similar materials. It can be seen from the overburden fracture evolution model established by theoretical calculations and similarity model experiments that the overlying rock layer’s breakage law is consistent. Additionally, the height of the “three zones” and the law of the fracture angle are basically consistent. Obtaining the synchronous collapse of the overlying strata controlled by the key strata, the interval of the upper key strata is larger than that of the lower key strata, and the mining interval is approximately double the size of the deformed rock height. According to the overburden movement, the distribution law of the overburden separation rate is obtained. The strain in the stress concentration area is negative, and when the stress is released suddenly, the strain increases rapidly. Fracture development is detected by the p-wave velocity in the model. Moreover, certain guidance for the horizon selection of high and low-level gas drainage roadways is provided by this study.

## Introduction

Coal is asignificant source of energy^1–3^, but the production process is often disastrous. It is very important for the mining face pressure control and the horizon selection of high- and low-level drainage roadways to explore the dynamic development law of overlying strata breakage. According to the traditional “three zones” theory^4–7^, when the mining face moves forward, the roof strata will hang out and then fail, forming a caving zone, a fractured zone and a bending subsidence zone. The “three zones” have their own characteristics of rock strata movement and deformation.

Many research results on mine pressure and rock strata movement have been reported; among them, the key stratum theory is the mainstream theory at present^8–13^. He *et al*.^14^ analyzed the mechanisms of mining tremors caused by key strata movement and instability. Sun *et al*.^15^ proposed a similar hyperbolic settlement model to describe the movement and failure of internal loads. Fan and Liu^16^ proposed a conceptual model of broken rock mass compaction by elastic theory. Luan *et al*.^17^ determined that the thickness and span of key strata have a more dominant effect on the stability of key strata compared with other factors, and the increase in uncertainty levels leads toa decrease in the stability probability. Liang *et al*.^18^ showed that in a fully mechanized mining face with a high mining height, the first subordinate key layer has two structural forms and six types of movement. Xie and Xu^19^ researched the key stratum effect on the mining abutment pressure of a coal seam. Han *et al*.^20^ presenteda method to calculate the working surface abutment pressure by key strata theory. Gao *et al*.^21^ demonstrated that the first fracture occurrence ofthe key layer increases the displacement and the stress and that the second fracture partially releases the stress.

Similarity model experiments are common methods for studying overburden. In recent years, much research has been done on the two-zone failure mode and related cracks in overlying strata on the working face of shallow thick seams^22–26^. Li*et al*.^27^ studied the impact of the compound breakage of key strata on the overburden movement and strata pressure behavior during fully mechanized caving mining in shallow and extremely thick seams based onUDEC numerical simulation software. Kang *et al*.^28^ created a large-scale similarity model experimentsusing a real case to simulate massive roof collapse during longwall coal retreating mining. Le *et al*.^29^ proposed a discontinuous modeling method to research longwall top coal caving behavior, including the stress distribution, coal and rock failures, top coal caving and roof strata rupture, and to analyze the impact of overlying strata movement on top coal caving. The similarity model experiments was used to calculate the formation and distribution of overlying stratacracksinduced by uplift mining^30^. Using the similarity model experiments, the movement laws of the overlying strata in the upper and lower strata were analyzed^31^. The development height of an air-conducting fracture zone in the gob overburdenwas verifiedthrougha similarity model experiments and numerical simulation of particle flow code software^32^. By means of a theoretical analysis and physical and numerical simulations, He *et al*.^33^ analyzed the motion boundary shapes of bedrock and unconsolidated formations, establishedthe motion boundary fitting equations for both, analyzed the influence of key formations on the formation motion boundary shapes, and determined the principle of setting protective coal pillars.The failure law of overlying strata and the development of the “three belts” were studied through similarity model experiments, and the failure mode and rock failure under the weak cement roof mining conditions of longwall top coal mining were analyzed^34^. Ju and Xu^35^ determined and defined three structural models via field observations, similarity model experiments and theoretical analyses of the first longwall face with a 7.0 m mining height;these modelswere affected by the relative position of key stratain the overlying strata. Zhou *et al*.^36^ used theoretical analysis and similarity model experiments methods and found that the fracture has two development cycles and two peaks. In the three-dimensional similarity model experiments, the deformation law of overburden caused by continuous coal seam mining was studied^37^. Yan *et al*.^38^ investigatedthe shaft deformation law under caving and backfill mining bythe similarity criteria of similarity model experiments.The stability evolution law of clay aquifers during mining of extremely thick coal seams was studied through similarity model experiments^39^. Ghabraie *et al*.^40^ investigated the characteristics of multi-seam subsidence by means of several sand-plaster physical models.

Similarity model experiments are commonly used to study overlying rocks. Previous studies mostly used a comparative analysis of similar experimental results with UDEC or FLAC3d simulation results, mostly analyzing fracture zones. Similar experimental models stratified less. In addition, there have been few experimental tests of similar materials in the literature; that is, cylinder sample experiments and little support for overburden breaking models based on theoretical calculations have been found. The analysis of collapse zones and analysis from each layer of the seam roof have rarely been seen. In this paper, the following objectives are presented: (1)Cylinder sample experiments are performed to provide a basis for the selection of similar materials. (2)Based on the on-site geological conditions, the overburden fracture evolution model ofthe mining face is established according to the calculation method of key strata theory, the fracture angle of layer theory and “three zones” theory. (3)The overburden breaking model established based on theoretical calculations verifies the accuracy of the similarity model experiments, determines the key strata level of overburden on the working face of deep thick seams, and obtains the distribution law and the breaking angle of the overlying strata. Additionally, the height of the caving zone and the fractured zone, the strain change law and the damage degree of the overburden are obtained.

It is necessary to arrange 3 roadways for the mining of the working face in a mine in Shanxi Province to solve the problem of gas drainage, which leads to complicated construction management conditions, a large amount of engineering work and an extremely difficult connection between mining and tunneling. A new method of high- and low-level gas drainage roadways is proposed to solve the problem of gas drainage. The method mentioned in this paper has certain guidance for the working face pressure control and the horizon selection of high- and low-level gas drainage roadways.

## Cylinder sample uniaxial compression test

The average thickness of the main mining 15# coal seam is 5.4 m, the average dip angle is 4°, and the average buried depth is 480 m. The daily mining distance of this working face is 6.4 m. There are two surface boreholes in the mining face, and the lithology and thickness of overburden rock on the mining face can be obtained by the comprehensive bar chart of the mine. The overburden rock parameters of the mining face are shown in Table 1.

**Table 1:** Overburden parameters.

No.	Lithology	*H*_*i*_/m	*h*_*i*_/m	*P*_*ai*_/MPa	*P*_*bi*_/MPa	No. ratio	*γ*_*i*_/(KN/m^3^)	*E*_*i*_/GPa	*R*_*i*_/MPa	${{\boldsymbol{\varphi }}}_{{\boldsymbol{i}}}$/°
38	Sandy mudstone	280	10	50	0.16	537	**25**	**15**	**10**	
37	gritstone	270	6	120	0.38	337	**25.3**	**28**	**12**	
36	Siltstone	264	8	90	0.28	355	**26**	**18**	**7**	
35	Sandy mudstone	254	6	52	0.16	537	**25**	**15**	**10**	
34	mid-stone	248	12	130	0.41	328	**25.5**	**25**	**11**	
33	Sandy mudstone	236	16	40	0.13	719	**25**	**15**	**10**	
32	Siltstone	220	12	90	0.28	355	**26**	**18**	**7**	
31	mid-stone	208	4	130	0.41	328	**25.5**	**25**	**11**	
30	Sandy mudstone	204	6	40	0.13	719	**25**	**15**	**10**	
29	Siltstone	198	6	90	0.28	355	**26**	**18**	**7**	
28	mid-stone	192	16	130	0.41	328	**25.5**	**25**	**10**	
27	Siltstone	176	8	90	0.28	355	**26**	**18**	**7**	
26	Sandy mudstone	168	6	40	0.13	719	**25**	**15**	**10**	
25	Fine sandstone	162	8	110	0.34	337	25.5	23	11	
24	Sandy mudstone	154	10	40	0.13	719	**25**	**15**	**10**	
23	mid-stone	144	8	120	0.38	337	**25.5**	**25**	**10**	
22	Sandy mudstone	136	10	40	0.13	719	25	16	11	
21	No. 8_1_ coal	126	1	10	0.03	1182	14	0.3	0.1	
20	Mudstone	125	3	40	0.13	719	**24**	**10**	**1**	
19	Siltstone	122	8	65	0.20	519	**26**	**18**	**7**	
18	No. 8_4_ coal	114	1	10	0.03	1182	**14**	**0.3**	**0.1**	
17	Sandy mudstone	113	4	40	0.13	719	**25**	**15**	**10**	
16	Fine sandstone	109	4	110	0.34	337	**26**	**20**	**10**	
15	No. 9 coal	105	1	10	0.03	1182	**14**	**0.3**	**0.1**	
14	Sandy mudstone	104	4	30	0.09	755	**25**	**15**	**3**	
13	Fine sandstone	100	14	100	0.30	346	**25.3**	**28**	**11**	
12	K4 Limestone	86	2	120	0.38	337	**26**	**40**	**18**	
11	Sandy mudstone	84	6	30	0.09	755	**25**	**15**	**3**	
10	gritstone	78	4	80	0.25	355	**25.3**	**28**	**12**	
9	mid-stone	74	12	75	0.23	437	**25.5**	**25**	**11**	**43**
8	Sandy mudstone	62	4	30	0.09	873	**25**	**15**	**3**	**38**
7	K3 Limestone	58	4	120	0.38	337	**26**	**40**	**18**	**45**
6	Fine sandstone	54	5	65	0.20	519	**26**	**20**	**10**	**42**
5	Sandy mudstone	49	8	20	0.06	773	**25**	**15**	**3**	**38**
4	K2 Limestone	41	5	120	0.38	337	**26**	**40**	**18**	**45**
3	Sandy mudstone	36	6	20	0.06	773	**25**	**15**	**3**	**38**
2	Fine sandstone	30	8	65	0.20	519	**26**	**20**	**10**	**42**
1	Sandy mudstone	22	4	20	0.06	773	**25**	**15**	**3**	**38**
0	No. 15 coal	18	5.4	10	0.03	1182	14	0.3	0.1	—
−1	Sandy mudstone	12.6	12.6	60	0.19	528	25	15	3	—

Note: *H*_*i*_—total thickness at the i-th layer; *h*_*i*_—thickness of layer *i*; *P*_*ai*_—prototype compressive strength of layer *i*; *P*_*bi*_—model compressive strength of layer *i*; *γ*_*i*_—body force of layer *i*; *E*_*i*_—elastic modulus of layer *i*; *R*_*i*_—tensile strength of layer *i*; *φ*_*i*_—friction angle of layer *i*.

### Experimental design

To obtain the right ratio of similar materials depending on the compressive strength of similar materials, a cylinder sample (i.e., 50 mm in diameter, 100 mm high) with sand, lime, gypsum and water was made. The No. ratio of the material is *AB*(10-*B*); the quality proportions of sand, lime and gypsum are *A*/(*A* + 1), *B*/[10(*A* + 1)] and (10 − *B*)/[10(*A* + 1)], respectively.

To optimize the number of cylinder samples, an orthogonal experiment was designed, taking the proportion of sand as the level and sand, lime and gypsum as the factors. The orthogonal design is shown in Table 2.

**Table 2:** Ratio level.

Level	1	2	3	4
Sand: Gels	3:1	5:1	7:1	9:1
Lime: Gypsum	1:9	3:7	5:5	7:3

**Figure 1:**
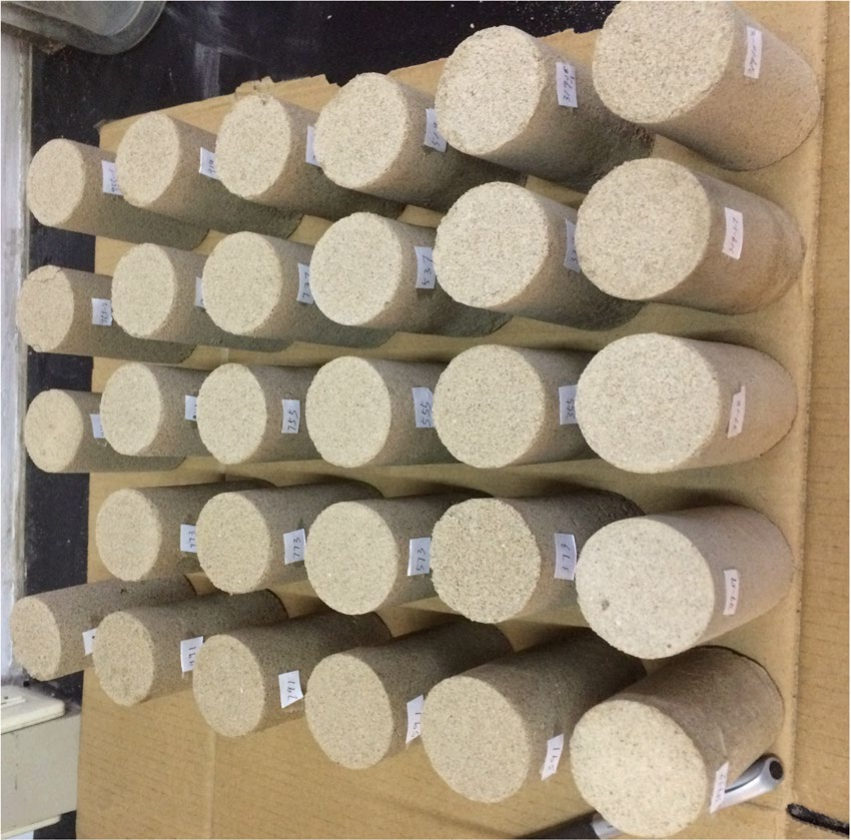
Cylinder sample model.

The volume of the cylinder sample is 196.35 cm^3^, and the mass is 314.16 g, according to the apparent density of 1.6 g/cm^3^. The water mass is 34.91 g according to 1/9th of the cylinder sample mass. The components were calculated according to the No. ratio, as shown in Table 3. The cylinder sample model is shown in Fig. 1.

**Table 3:** Compressive strength corresponding to the No. ratio.

No. experimental	No. ratio	Mass of sand/g	Mass of lime/g	Mass of gypsum/g	Compressive strength/MPa
1	319	235.62	7.854	70.686	0.473
2	337	235.62	23.562	54.978	0.368
3	355	235.62	39.27	39.27	0.251
4	373	235.62	54.978	23.562	0.14
5	391	235.62	70.686	7.854	0.083
6	519	261.8	5.236	47.124	0.203
7	537	261.8	15.708	36.652	0.144
8	555	261.8	26.18	26.18	0.107
9	573	261.8	36.652	15.708	0.074
10	591	261.8	47.124	5.236	0.052
11	719	274.89	3.927	35.343	0.137
12	737	274.89	11.781	27.489	0.112
13	755	274.89	19.635	19.635	0.087
14	773	274.89	27.489	11.781	0.062
15	791	274.89	35.343	3.927	0.041
16	919	282.74	3.142	28.274	0.076

### Experimental results

**Figure 2:**
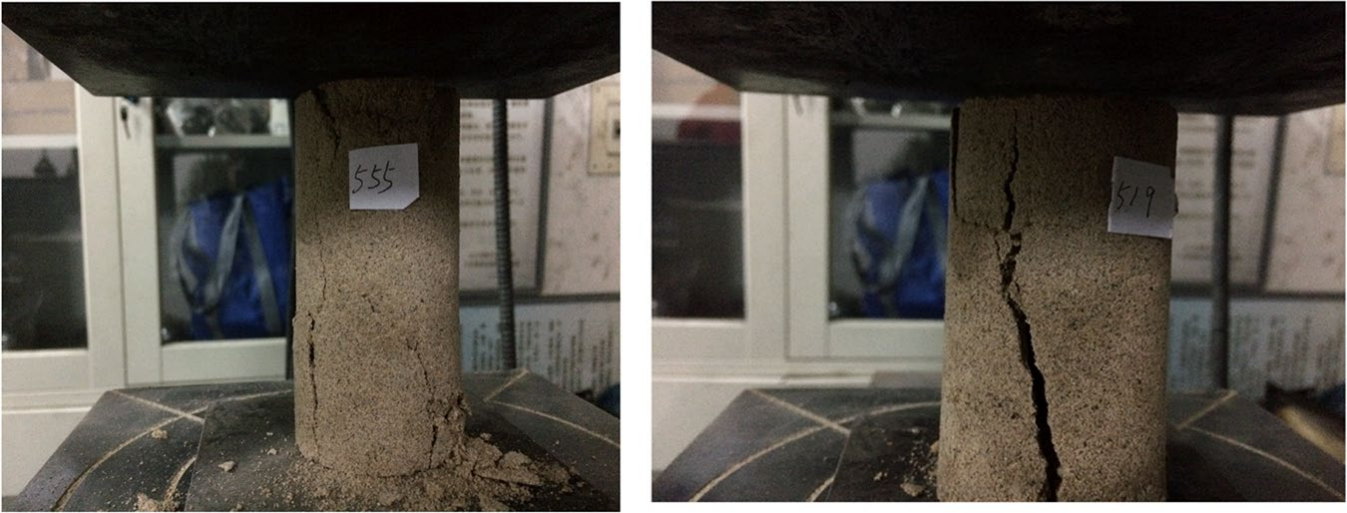
Cylinder sample fracturing process.

A uniaxial compression test was conducted with a universal servo testing machine to test the compressive strength of the cylinder sample. The experimental process is shown in Fig. 2. The compressive strength obtained from the test is shown in Table 3.

**Figure 3:**
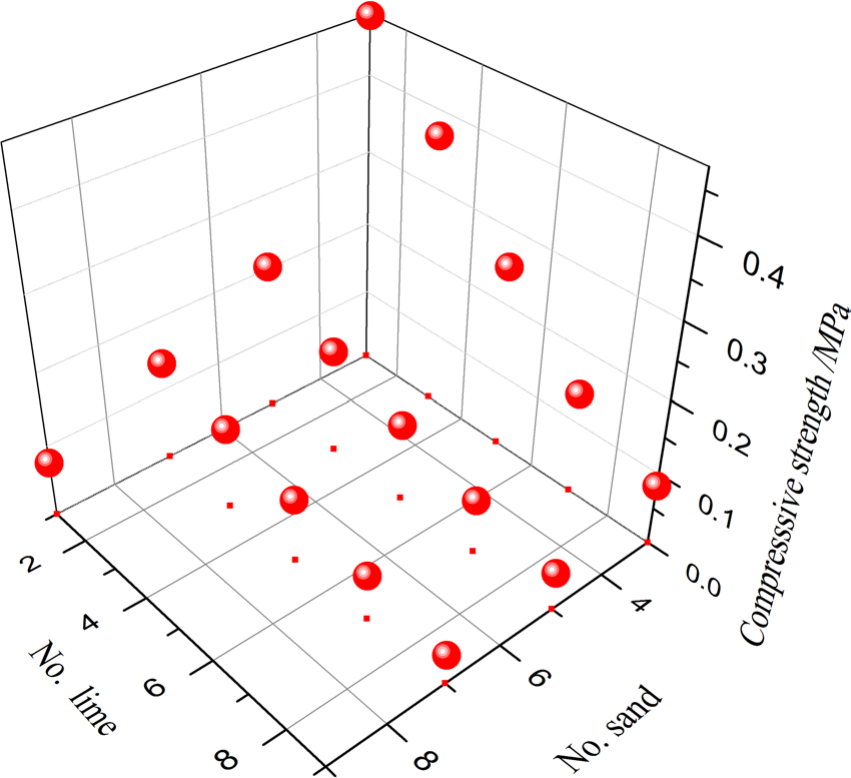
The relationship between the No. ratio and compressive strength.

The sum of the No. ratios of lime and gypsum is 10. The compressive strength of the cylinder sample is a function of the No. ratio of sand and lime, where the No. ratios of sand and lime are independent variables, and their relation is shown in Fig. 3.

**Figure 4:**
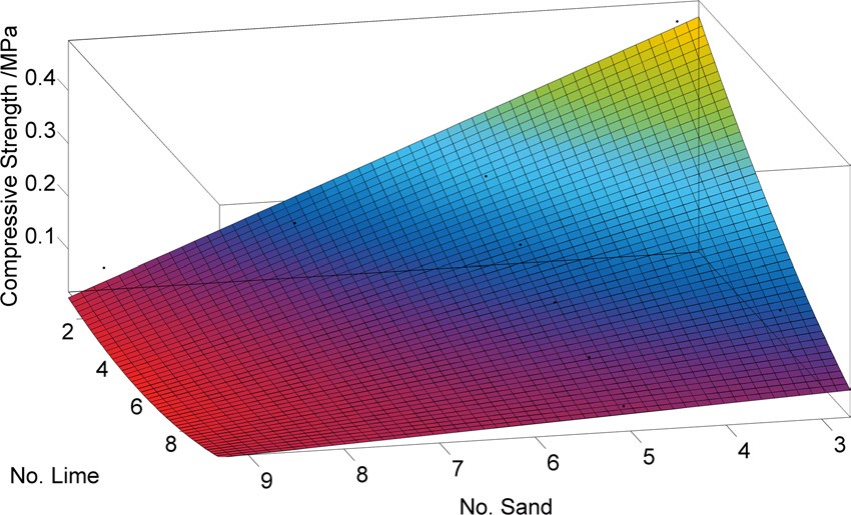
Fitting result.

The compressive strength of the cylinder sample decreases with an increasing No. ratio of sand and lime. With an increase in the No. ratio of sand, the influence of the No. ratio of lime on the compressive strength of the cylinder sample decreases. In parallel, with an increase in the No. ratio of lime, the influence of the No. ratio of sand on the compressive strength of the cylinder sample decreases. The No. ratio of sand and lime are independent variables *x* and *y*, respectively, and compressive strength is the dependent variable *f(x, y)*. Using MATLAB to select the polynomial fit for the fitting, the fitting formula is shown as Eq. (1), and the fitting diagram is shown in Fig. 4.

1 $$\begin{array}{ccc}f(x,y) & = & 1.928-0.7326x\,-\,{\rm{0.1455y}}+0.1013{x}^{2}+0.0412xy\\  &  & -\,0.004708{x}^{3}-0.003163{x}^{2}y\,{{\rm{R}}}^{2}=0.9924\end{array}$$

In Eq. (1), *x* is an integer ranging from 3~9, and *y* is an integer ranging from 1~9, from which the No. ratio of the desired compressive strength can be obtained, and the No. ratio of similar materials corresponding to the compressive strength of rocks can be obtained according to the similarity ratio, as shown in Table 1.The parameters used in the following are listed in Table 1.

## Overburden theoretical calculation model

### Calculation of the overburden “three zones”

According to the theory of mine pressure control^41,42^, the maximum height of the caving zone of the general working face is calculated by formula (2) when the overburden is medium hard. In the hard overburden case, it can be calculated by formula (3):

2 $${H}_{c}=\frac{100M}{4.7M+19}\pm 2.2=9.97 \sim 14.37$$

3 $${H}_{c}=\frac{100M}{2.1M+16}\pm 2.5=17.25 \sim 22.25$$

Where *Hc* is the height of the caving zone, m; and *M* is the mining height of the coal seam, 5.4 m.

The upper part of the coal seam is a thinner mudstone, the upper part of the mudstone is a thicker fine sandstone, and the upper part of the thicker fine sandstone is a hard limestone. The whole part is between medium hard and hardrock; therefore, the height of the caving zone is 14.37~17.25 m.

The maximum height of the fractured zone is calculated by formula (4) when the overburden is hard:^43^

4 $${H}_{f}=\frac{100M}{1.2M+2}\pm 8.9=54.8 \sim 72.6$$

Where *H*_*f*_ is the height of the fractured zone, m.

Because the overburden has more limestone and sandstone, the whole is hard rock, so the maximum height of the fractured zone is 54.8~72.6 m.

### Calculation of overburden breakage

According to the key stratum theory calculation formula^44,45^, the parameter to obtain the load *q*_1_ of the first layer itself is:

5 $${q}_{1}={\gamma }_{1}\ast {h}_{1}=25\ast 4=100\,{\rm{KPa}}$$

Considering the effect of the second layer on the first layer, the load on the first layer is:

6 $${({q}_{2})}_{1}=\frac{{E}_{1}\ast {{h}_{1}}^{3}({\gamma }_{1}\ast {h}_{1}+{\gamma }_{2}\ast {h}_{2})}{{E}_{1}\ast {{h}_{1}}^{3}+{E}_{2}\ast {{h}_{2}}^{3}}=26.4\,{\rm{KPa}}$$

Where*(q*_*m*_)_*n*_ represents the load on the n layer when the rock at the m layer above the n layer is calculated, KPa.

The effect of the second layer on the first layer should not be considered by reference to the key stratum theory, because the second layer is strong and thick and has no effect on the first layer, so the first layer is subjected to a load of 100 KPa.

Similarly, *q*_2_ = 208KPa, *(q*_2_)_2_ = 272.8KPa, *(q*_3_)_2_ = 271KPa, the load on the second layer is 272.8KPa, and the upper strata are controlled by it.

Similarly, *q*_4_ = 130 KPa, *(q*_2_)_4_ = 130.1KPa, *(q*_3_)_4_ = 151.5KPa, *(q*_4_)_4_ = 159 KPa, *(q*_5_)_4_ = 177.5KPa, *(q*_6_)_4_ = 78.4KPa, the load on the fourth layer is 177.5KPa, and the upper four strata are controlled by it.

Similarly, *q*_9_ = 306 KPa, *(q*_2_)_9_ = 391 KPa, *(q*_3_)_9_ = 499 KPa, *(q*_4_)_9_ = 540.3 KPa, *(q*_5_)_9_ = 315 KPa, so the loading on the ninth layer is 540.3 KPa, and the upper three layers are controlled by it.

Based on the theory of clamped-clamped beams in material mechanics^45,46^, when the maximum normal stress at both ends of the beam is equal to the tensile strength of rock, the overburden will be broken for the first time. The periodic interval of roof breaking is calculated by reference to the cantilever theory of material mechanics^47^. The first interval of roof breaking of layer i is recorded as *(L*_*a*_)_*i*_, and the period is recorded as *(L*_*b*_)_*i*_. The formula is as follows:

7 $${({L}_{a})}_{i}={h}_{i}\ast \sqrt{\frac{2{R}_{i}}{{q}_{i}}}$$

8 $${({L}_{b})}_{i}={h}_{i}\ast \sqrt{\frac{{R}_{i}}{3{q}_{i}}}$$

It is estimated that *(L*_*b*_)_*i*_ = *(L*_*a*_)_*i*_/2.45.

The mechanical derivation angle of the maximum principal stress and horizontal stress can be obtained as follows:${{\rm{\theta }}}_{1}=(1/2){\tan }^{-1}(3L/h)$, where *L* represents the suspended span length of the beam, m, and *h *represents the thickness of the key stratum, m.

The suspended span length of the rock beam is always larger than the height of the rock beam, and 3 *L*/*h* is larger than 3, thus $45^\circ  > {\theta }_{1} > 35^\circ$.

According to the Mohr-Coulomb criterion^48,49^, the angle between the rock damage plane and the maximum principal stress is ${\theta }_{2}=45^\circ -\varphi /2$, where $\varphi$ represents the angle of the internal friction of the rock, °.

If the friction angle of sandstone and limestone is generally 20°~60°, then $35^\circ  > {\theta }_{2} > 15^\circ$.

The calculation formula of the included angle between the rock damage plane and the horizontal plane is as follows:

9 $$\beta =45^\circ -\frac{\varphi }{2}+\frac{1}{2}{\tan }^{-1}\frac{3L}{h}$$

Where $\beta$ is the fracture angle, °, and $80^\circ  > {\rm{\beta }} > 50^\circ$. The first fracture angle of layer *i *is recorded as ${({\beta }_{a})}_{i}$. The periodic fracture angle of layer *i *is recorded as ${({\beta }_{b})}_{i}$.

Through the analysis of the formula of the roof breaking interval of rock, it can be concluded that the length and thickness of the rock beam and the rock shape have little influence on the rock fracture angle, which is only approximately 10°. In addition, the property of the rock friction angle has a great influence on the rock fracture angle.

According to the theoretical formula, the roof breaking interval and fracture angle of each stratum can be calculated, as shown in Table 4:

**Table 4:** Roof breaking interval and the fracture angle of key stratum.

No.	(L_a_)_i_/m	(L_b_)_i_ /m	(β_a_)_i_/°	(β_b_)_i_/°	Control height/m
1	31	12.6	69.4	68	4
2	68.5	28.3	67.4	66.3	14
4	71.2	29.1	66.8	65.9	28
9	76.6	31.3	67.4	66.3	22

The periodic the roof breaking interval of key strata is less than the first roof breaking interval. According to the calculation formula of the fracture angle of key strata, the periodic fracture angle is less than the first fracture angle.

If the roof breaking interval of the lower strata whose is smaller than that of the upper strata, then the lower strata is the key strata, with reference to the key strata theory. Therefore, it can be determined that the first, second and fourth strata are the key strata.

According to the control height of the key strata and the theoretical calculation results of the “three zones” of overburden, the height of the caving zone is the control height of the second key strata (18 m), and the height of the fractured zone is the control height of the ninth strata (68 m).

### Establishment of overburden fracture evolution model

**Figure 5:**
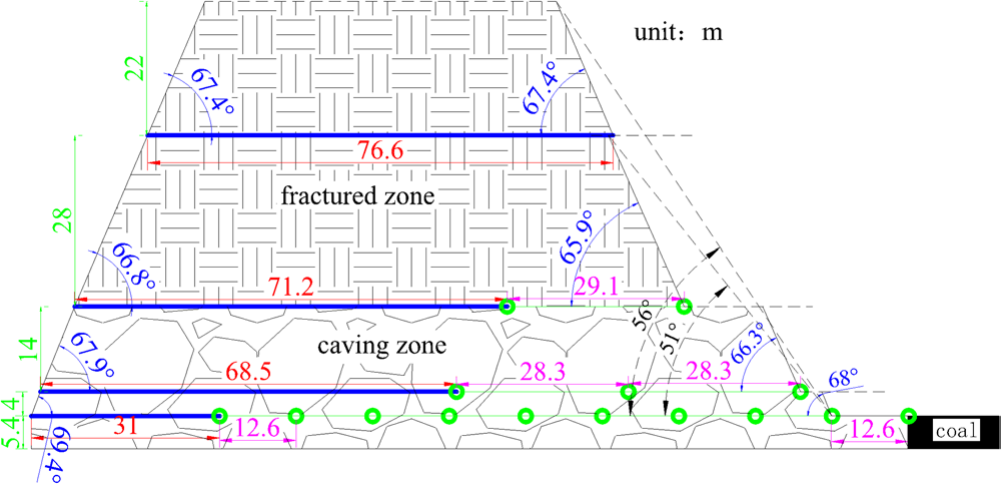
Overburden fracture evolution model.

Based on the on-site geological conditions, the overburden fracture evolution model of the mining face is established according to the calculation method of the key strata theory, the fracture angle of layer theory and the “three zones” theory, as shown in Fig. 5.

The blue line in the figure is the first roof breaking interval of the key stratum floor, which is made at the boundary according to the first fracture angle and the control height of the key stratum. The green line in the figure is the periodic roof breaking interval of the key stratum floor, which is made according to the connection between the periodic fracture angle and the control height of the key stratum. The overburden fracture evolution model shows that the overall whole fracture angle of overburden at the open-off cut side is basically the same as the rock fracture angle. The whole fracture angle of overburden at the mining side is greatly affected by the roof breaking interval, breaking position and fracture angle of the lower strata, and its value fluctuates greatly, with an average value less than the periodic fracture angle of the rock. The periodic fracture angle and roof breaking interval of the first key stratum of the coal seam roof are affected by the mining speed, while the periodic roof breaking interval of the upper key stratum is affected by the periodic roof breaking interval, the fracture angle and the position of the lower key stratum.

## Similarity material model experiment

### Model establishment

The similarity material model experiment is based on the two-dimensional test bed of the laboratory of China University of Mining and Technology (Beijing). The length, width and height of the test bed are 3000 mm, 250 mm and 2000 mm respectively. According to the similarity principle^50^, the geometric similarity ratio between the entity and the model is set as 200, the time similarity ratio is set as 14.14, the gravity similarity ratio is set as 1.6, and the stress similarity ratio is set as 320.

**Figure 6:**
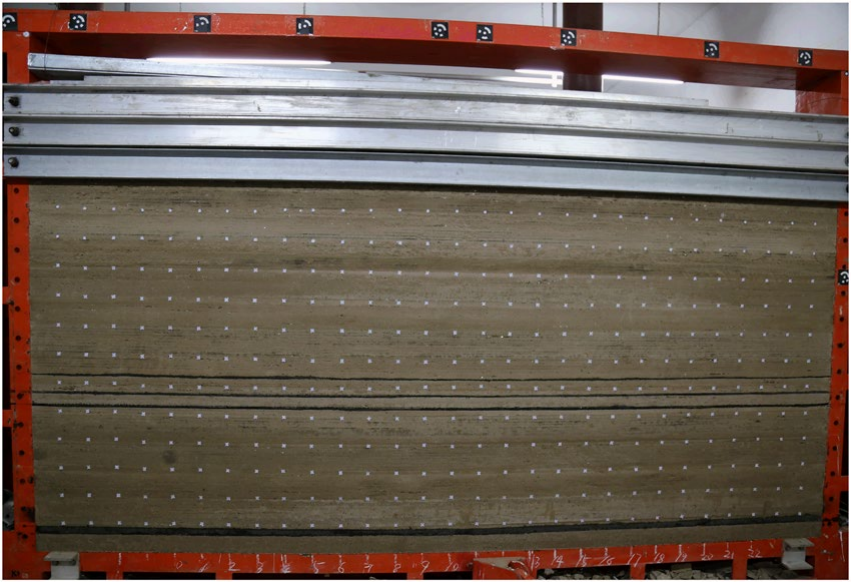
Experimental model.

The displacement observation points and strain monitoring points were set in the experimental model. The similar materials were sand, lime, gypsum and water, and the model materials were proportioned according to the similarity ratio.The No. ratios of each layer are shown in Table 1. Mica slices were placed between adjacent layers to simulate stratification. The physical model was constructed layer by layer with a thickness of approximately10 mm and a total thickness of 1.4 m.The completed physical model was allowed to dry naturally. The physical model after removing the mold is shown in Fig. 6.

The range of the model simulates: 12.6 m below the coal floor, 480 m above the roof and a 5.4 m thick coal seam on average. The height of the simulated is approximately 498 m, the height of the simulated material is approximately 280 m, and the remainder of the height is only simulated to the surface with a counterweight of approximately 218 m.

### Mining process

**Figure 7:**
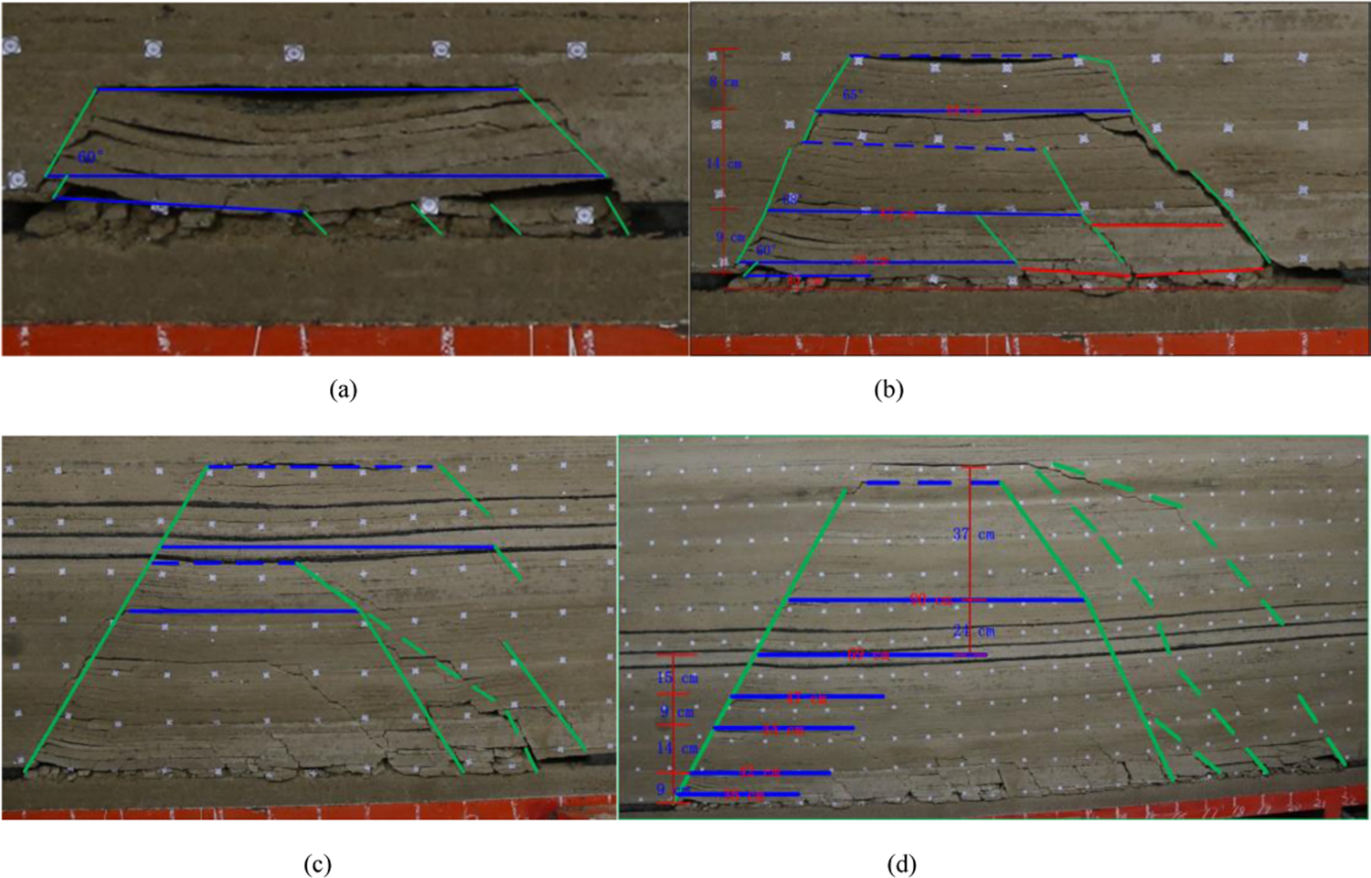
Overburden evolution process.

In the model, the displacement, strain, fracture angle, interval of roof collapse and height of roof collapse development were recorded for each mining 5 cm. The model status at mining depths of 43 cm, 85 cm, 115 cm and 220 cm is shown in Fig. 7(a–d).

In Fig. 7(a–d), the blue horizontal line is the first interval of roofing breaking of the key stratum floor, and the interval is made at the boundary according to the first fracture angle and the control height of the key stratum. The red vertical line is the control height of the key stratum, the blue dotted line is the control height of the key stratum at the first breakage, and the green oblique line is the rock stratum fracture angle. A total of 220 cm is mined in a similar model, and there are 7 layers of fractures in the key overburden strata above the roof of the coal seam. The roof breaking interval are 20 cm, 38 cm, 42 cm, 44 cm, 47 cm, 69 cm and 90 cm. The control heights of the fractures are 2 cm, 7 cm, 14 cm, 9 cm, 15 cm, 24 cm and 37 cm.

**Figure 8:**
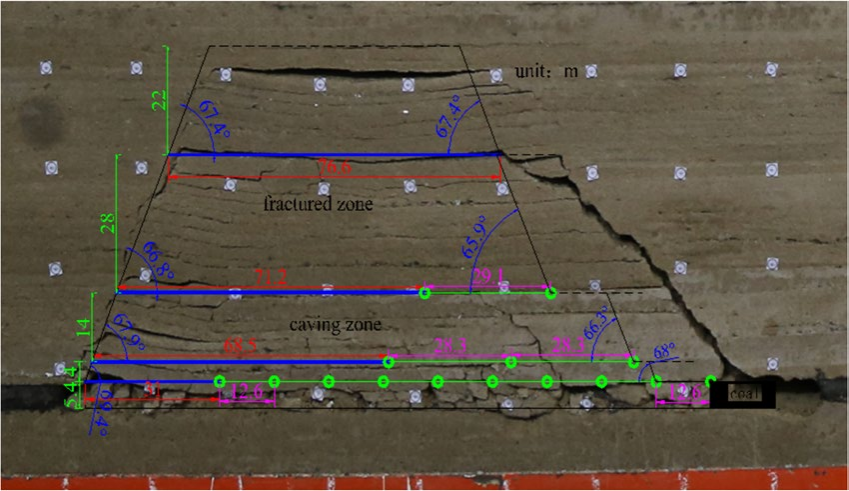
Comparison of experimental and theoretical models.

When the model was mined 85 cm, four key strata were broken. A comparison with the theoretical calculation model is shown in Fig. 8. The overall shapes of the theoretical calculation model and experimental model are basically the same. Due to the stratification complexity of the experimental model, the breakage position of the lower strata affects the upper strata, the fracture angle of the experimental model is slightly less than the theoretical calculated value, the control height of the key strata in the experimental model is basically consistent with the theoretical calculated value, and the roof breaking interval of the experimental model is slightly larger than the theoretically calculated value.

### Collapse law of the model

**Figure 9:**
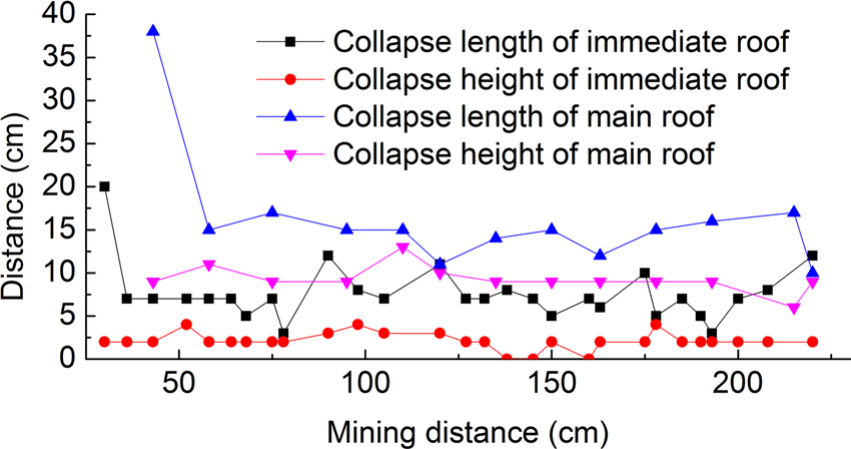
The relationship between the mining distance and collapse.

The collapse length and height of the immediate and main roof were measured using millimeter steel rulers in the experiment. Figure 9 shows the length and height of overburden collapse during mining.

The average value of the collapse height of the immediate roof was 2.11 cm, the first interval of roofing breaking was 20 cm, and the periodic interval mean of roofing breaking was 7.11 cm. The average collapse height of the main roof is 9.31 cm, the first interval of roofing breaking is 38 cm, and the average of the periodic interval of roofing breaking is 14.33 cm. The collapse length of the main roof was roughly twice as long as that of the immediate roof. The height of the caving zone is basically 9 cm high, and the actual height is 18 m due to the model scale of 200.

**Figure 10:**
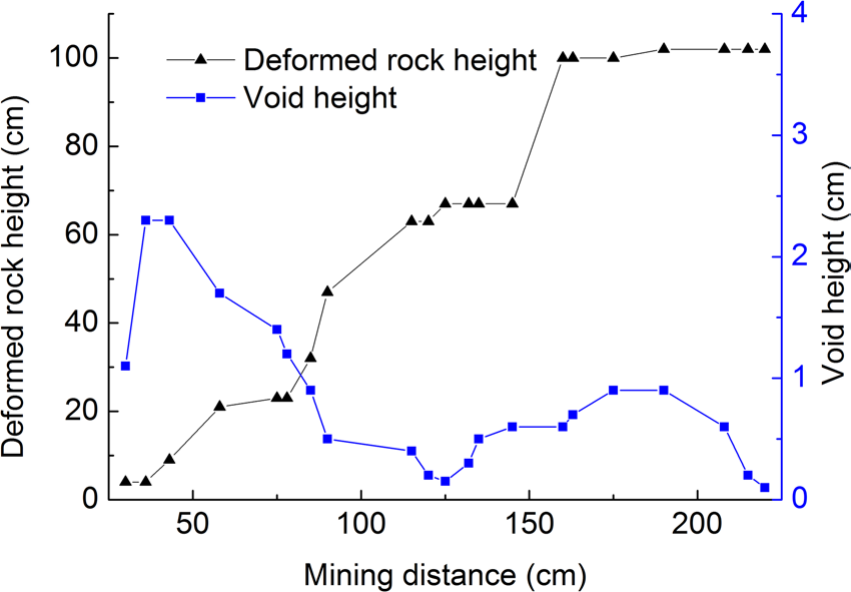
The relationship between the mining distance and deformed rock height and void height.

The maximum vertical distance between the strata with obvious movement and deformation during the process of mining and the roof of the coal seam is called the deformed rock height. The vertical distance between the highest strata with movement and the upper strata without movement is called the void height. During the mining process, the relation between the mining interval and deformed rock height and void height is shown in Fig. 10.

The mining interval is roughly twice the deformed rock height. Due to the synchronous deformation of the rock strata at a certain height controlled by the key strata, the deformed rock height will jump up after stabilizing for a certain interval during the mining process of the working face. With the mining of the working face, the void height gradually decreases after reaching the maximum value, and the amplitude gradually decreases and reaches the minimum value. The void height rises slightly after the goaf is re-compacted. It is speculated that the void height will change periodically with the deformed rock height, and the change side value is not large.

**Figure 11:**
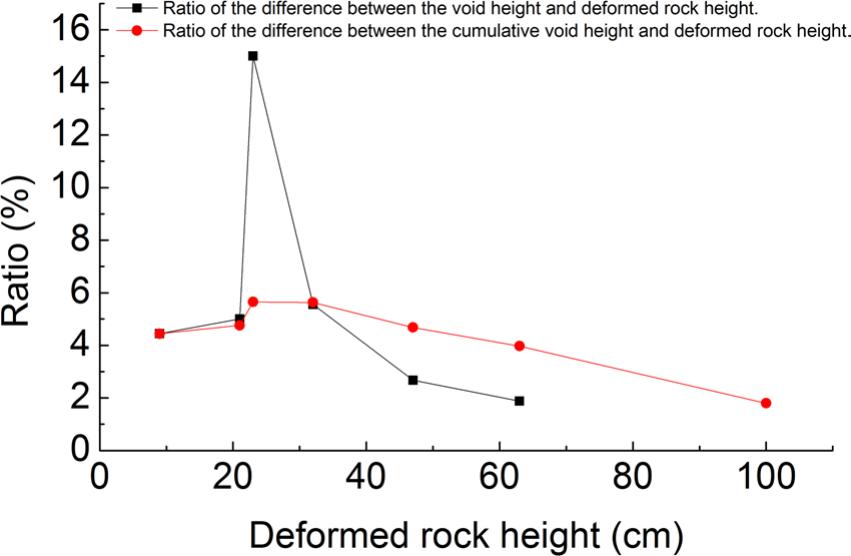
The relationship between the deformed rock height and ratio.

The ratio of the void height difference and the deformed rock height difference has a positive correlation with the dilatancy of the overburden. In addition, the ratio mentioned in the previous section and the ratio of the accumulated void height difference and the deformed rock height difference in the mining process were analyzed, as shown in Fig. 11.

When the mining interval is 85 cm, the deformed rock height is 32 cm; when the mining interval is 90 cm, the deformed rock height is 47 cm. The relationship between the deformed rock height and the ratio of the void height difference and deformed rock height difference was analyzed. When the deformed rock height is 32 cm, the ratio is relatively large; when the deformed rock height reaches 47 cm, the value decreases rapidly and then decreases slowly. It can be concluded that when the overburden is 32 cm high, the rock has a relatively large dilatancy, and its fractures are also developed. When the overburden is higher, the overburden has a relatively small dilatancy, and its fractures are not fully developed. It can be inferred that the upper limit of the fractured zone height is 32 cm, and the actual height is 64 m due to the model scale of 200. The dilatancy and fracture development of overlying strata are in direct proportion to the difference in the deformed rock height in the overlying strata, and the difference in the void height attenuates with mining, indicating that the dilatancy of the overlying strata decreases in the direction of the height.

### The displacement of overburden

In the model, displacement observation points begin to be arranged at a height of 1 cm above the coal seam, with a horizontal interval of 10 cm. There are 29 displacement observation points in each layer, and the number of displacement observation points in the first layer is A01, A02, A03…, A29. One layer of displacement observation point is arranged 10 cm high at every interval, and 12 displacement observation points are published. The serial numbers are A, B, C…, L, and the displacement observation point has a 12*29 grid shape.

**Figure 12:**
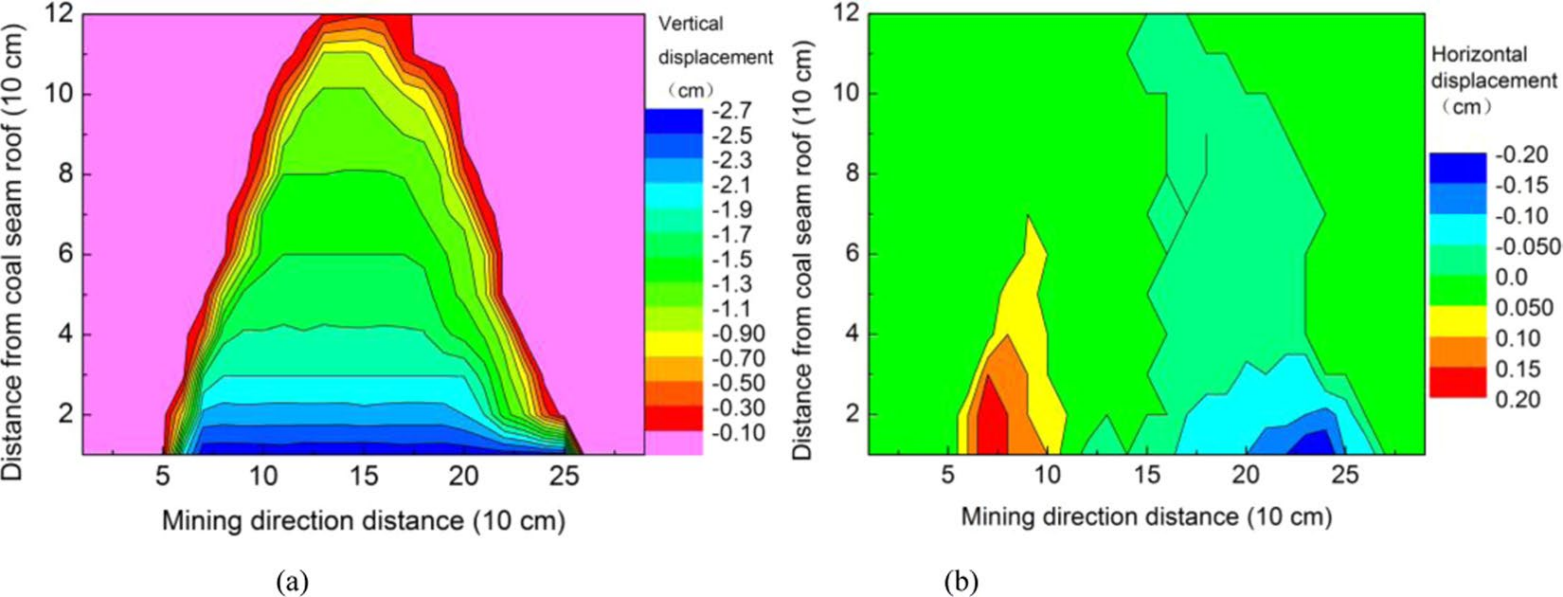
Distribution of the overburden displacement.

**Figure 13:**
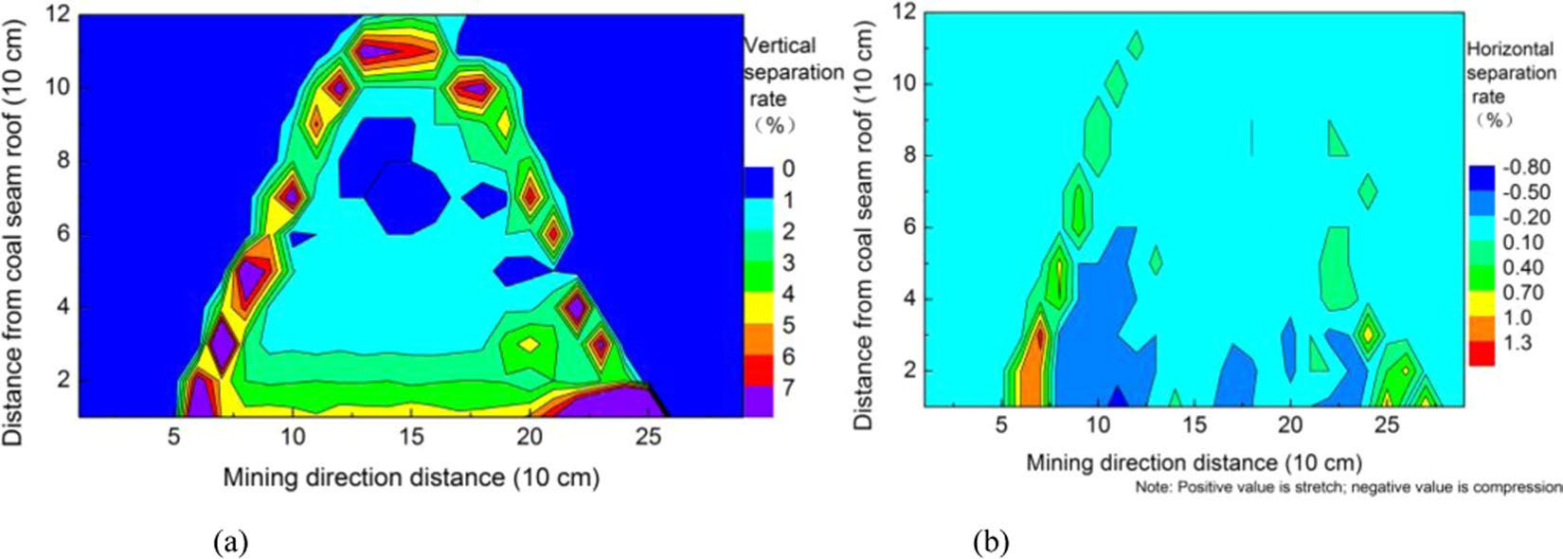
Distribution of the overburden separation rate.

After the model is dried, displacement observation points are arranged. To facilitate the recording and processing of displacement data, grids are arranged from bottom to top and from left to right (from line to column (i, j)). The Electronic Total Station was used to measure each displacement observation point successively before mining, and the displacement observation point was measured again when mining to 220 cm. The vertical displacement and horizontal displacement distribution of the overburden were obtained by comparing the two displacement observation points, as shown in Fig. 12(a,b), respectively. The distribution of the overburden separation rate was obtained by calculating the displacement difference and the distance between two points. The ratio of the vertical displacement difference and the vertical distance between two points and the ratio of the horizontal displacement difference and the horizontal distance between two points are shown in Fig. 13(a,b), respectively.

In coal seam mining, the overburden strata mainly subsided, the horizontal displacement was very small, and the vertical displacement of the overlying strata all sank within the influence range. The closer to the coal seam, the greater the subsidence is and the larger the influence scope is, and the vertical displacement is attenuated when it develops toward the height. The vertical displacement change in the overlying strata on the side of the open-off cut is relatively uniform, and void development is stable. The vertical displacement change in the overlying strata on the mining side is uneven, and the development of voids is unstable.

During the process of coal seam mining, the horizontal displacement of the overlying strata tends to move toward the middle in the affected area; that is, the overlying strata on the side of the open-off cut move toward the mining direction, the overlying strata on the side of the mining move toward the open-off cut direction, and the upward part moves less. Horizontal displacement attenuates in the direction of height development, which indicates that the void and fracture in the overburden rock layer closer to the coal seam are more developed. The horizontal displacement at both ends of the open-off cut side and mining side is larger, which indicates that the void and fracture here are more developed.

The height of 10 cm with a vertical displacement greater than 2.3 cm and the height of 10 cm with a vertical separation rate greater than 4% can be judged as a caving zone with a height of 10 cm. The height of 30 cm with a vertical displacement greater than 1.9 cm and the height of 30 cm with a vertical separation rate greater than 2% can be judged as a fractured zone with a height of 30 cm. The comprehensive analysis shows that the width of the “O” ring is approximately 20 cm.

The dilatancy and fracture development of the overlying strata are positively correlated with the displacement and separation rate of the overlying strata, and the displacement and separation rate decrease with the distance from the roof of the coal seam, indicating that the dilatancy and fracture of the overlying strata decrease in the height direction. The displacement and separation rate at both ends are relatively large, that is, the “O” ring, indicating that at the same level, the fractures at both ends of the open-off cut and mining side are more developed.

### Overburden fracture angle

**Figure 14:**
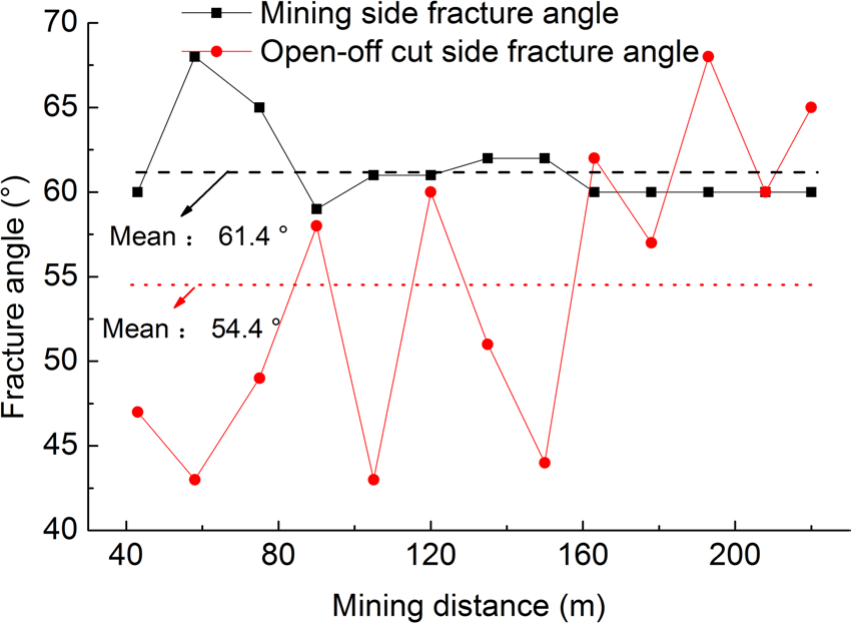
Relationship between the fracture angle and mining distance.

During the process of model mining, the fracture angles on both sides of the overburden were measured bya protractor every time the overburden collapsed. The whole fracture angles on the open-off cut side and the mining side are statistically analyzed when the miningare 43 cm, 58 cm, 75 cm, 90 cm, 105 cm, 120 cm, 135 cm, 150 cm, 163 cm, 178 cm, 193 cm, 208 cm and 220 cm. The results are shown in Fig. 14.

The whole fracture angle of the experimental model is consistent with the theoretical analysis. That is, the whole fracture angle of the overburden at the open-off cut side is basically consistent with the rock fracture angle, and the whole fracture angle of overburden at the mining side fluctuates greatly, whose mean is less than the rock periodic fracture angle.

During mining, the whole fracture angle at the open-off cut side fluctuates very little, with a mean of 61.4°. The whole fracture angle at the mining side fluctuates periodically with the mining of the working face, which is generally smaller than that at the open-off cut side and floats at a mean of 54.4°. The fracture at the side of the open-off cut develops steadily and upward, and the variation amplitude of fracture at the mining side is relatively large. The fracture on the mining side experienced the development and closure of the fracture during the process of upward development. On the whole, the development of the fracture at the side of mining is smaller than that on the side of the open-off cut.

### Strain at monitoring point

In the model, a resistance strain gauge was arranged in the middle of k2 limestone at the total height of the model at 19 cm and a height of 10 cm of coal seam roof. The position 50 cm away from the model boundary was seen as the open-off cut. The first strain monitoring point was arranged at the position of −10 cm on the side of the open-off cut, and a total of 10 strain observation points were arranged at a horizontal interval of 20 cm. The 10th strain observation point was located 170 cm from the open-off cut. The resistance strain gauge buried in the experimental model was connected to a laptop through a TCD-2A resistance strain collector, and the strain values were displayed on the laptop through supporting software.

**Figure 15:**
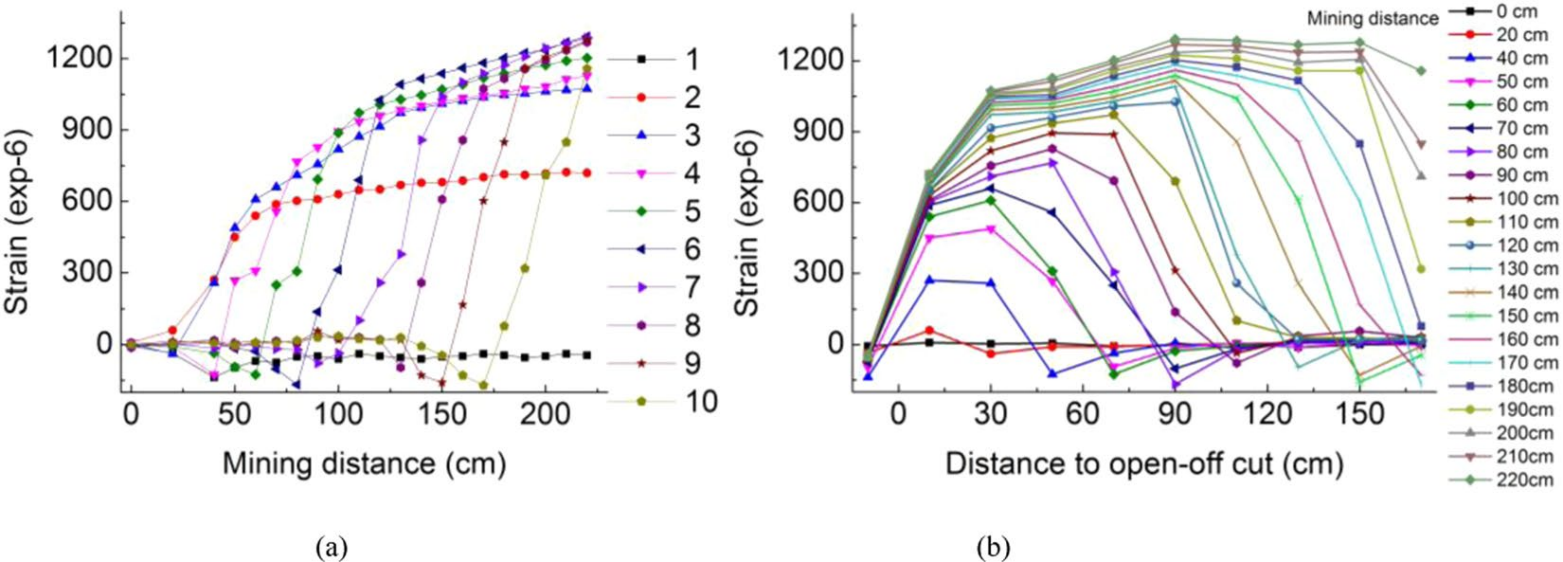
Relationship between the strain and mining distance.

Before mining, the value at each strain point was measured and recorded every 10 cm mined. The change in strain value at each single point with mining distance and strain distribution at each point at different mining distances are shown in Fig. 15(a,b), respectively.

The strain value of each strain monitoring point decreased slightly after mining, then increased rapidly, and finally tended to be stable. Due to the concentration of stress at both ends of the goaf during the process of mining, a small negative value of strain appeared, the stress inside the goaf was released suddenly, and the strain increased rapidly.

### Overburden damage zone

The model was tested by the rock acoustic wave parameter tester HS-YS4A. The test adopted a voltage of 160 V, a frequency of 100 Hz, a single channel straight through, a sampling size of 32 k, a sampling interval of 0.05 µs, an attenuation factor of 4 times, and a pulse width of 30 µs.

**Figure 16:**
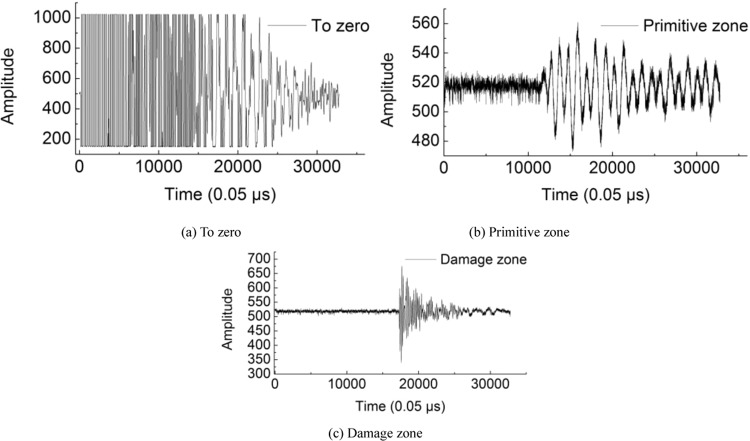
The P-wave shape.

The length of the test model is 25 cm, and the p-wave velocity is obtained from the measured time and distance. Figure 16(a–c) hows the p-wave waveform measured for zero, the primitive zone and the damage zone. The measured p-wave time is 2.6 µs for zero, 564.0 µs for the primitive zone and 867.8 us for the damage zone.

It can be analyzed from the measurement that the p-wave velocity spreads the fastest in the primitive zone and the slowest in the damage zone, and the p-wave amplitude and rate in the damage zone is obviously larger than those in the primitive zone. Therefore, the measurement of the p-wave velocity can be used to detect fracture development in the model.

The variation in the p-wave velocity before and after rock damage can characterize the damage value, and the calculation formula is as follows:

10 $$D=1-{\left(\frac{{v}_{1}}{{v}_{0}}\right)}^{2}$$

Where *D* represents the damage value of rock, *v*_0_ represents the p-wave velocity of the primitive zone, m/s, and *v*_1_ stands for the p-wave velocity of rock after damage, m/s.

The damage value of the rock in the model is 0.568.

## Conclusions

The overburden fracture evolution model obtained by theoretical calculation is consistent with the overburden breaking law obtained by the model experiment, and the two “three zones” are basically the same height. The overburden fracture angle and roof breaking interval control the overburden collapse, and the obtained overburden separation rate distribution provides theoretical guidance for the horizon selection of high- and low-level gas drainage roadways.

1. The cylinder sample model is made with sand, lime, gypsum and water, and the equation of compressive strength and No. ratio are fitted through a test of the compressive strength, and the No. ratio of similar materials corresponding to the compressive strength can be obtained.
2. According to the control height of the key strata and “three zones” theoretical calculation results of the overburden, the height of caving zone is the control height of the second key strata, 18 m, and the height of fractured zone is the control height of the ninth stratum, 68 m. From the overburden fracture evolution model established based on the theoretical calculation, it can be concluded that the whole fracture angle of the overburden at the open-off cut side is basically consistent with the rock fracture angle, and the whole fracture angle of overburden at the mining side fluctuates greatly, whose mean is less than the rock periodic fracture angle.
3. The similarity model is established to obtain the key strata controlling the synchronous collapse of the upper strata, and the roof breaking interval of the upper key strata is larger than that of the lower key strata, with the similarity principle. The rock collapse in the caving zone is periodic, and the caving height is 9 cm. According to the deformed rock height and the void height in the mining process, the upper limit of the height of the fractured zone is 32 cm, and the mining interval is approximately twice the deformed rock height.
4. The distribution law of the overburden separation rate is obtained according to the overburden displacement, and the height of the caving zone is 10 cm, the height of the fractured zone is 30 cm, and the width of the “O” ring is 20 cm. The whole fracture angle of the overburden in the experimental model is smaller than that of the rock strata. The whole fracture angle at the open-off side fluctuates very little with the advancement of the mining process, with a mean of 61.4°. The whole fracture angle on the mining side fluctuates at a mean of 54.4° with the mining of the working face.
5. Due to the stress concentration at both ends of the goaf during the mining process, the strain appears to have a small negative value, the stress inside the goaf is suddenly released, and the strain increases rapidly. The p-wave velocity spreads quickly in the primitive zone and slowly in the damage zone, and the p-wave amplitude and rate in the damage zone is significantly larger than that in the primitive zone. Therefore, the p-wave velocity can be measured to detect the fracture development in the model, and the damage value of the rock in the model is 0.568.

## Data Availability

The primary data used to support the findings of this study are available from the corresponding author upon request.
